# Semantic similarity in the biomedical domain: an evaluation across knowledge sources

**DOI:** 10.1186/1471-2105-13-261

**Published:** 2012-10-10

**Authors:** Vijay N Garla, Cynthia Brandt

**Affiliations:** 1 Yale Center for Medical Informatics, Yale University, 300 George Street, Suite 501, New Haven, CT 06520-8009, USA; 2 Connecticut VA Healthcare System, Bldg. 35A, Room 213 (11-ACSLG), 950 Campbell Avenue, West Haven, CT 06516, USA

**Keywords:** Semantic similarity, Information content, Information theory, Biomedical ontologies

## Abstract

**Background:**

Semantic similarity measures estimate the similarity between concepts, and play an important role in many text processing tasks. Approaches to semantic similarity in the biomedical domain can be roughly divided into knowledge based and distributional based methods. Knowledge based approaches utilize knowledge sources such as dictionaries, taxonomies, and semantic networks, and include path finding measures and intrinsic information content (IC) measures. Distributional measures utilize, in addition to a knowledge source, the distribution of concepts within a corpus to compute similarity; these include corpus IC and context vector methods. Prior evaluations of these measures in the biomedical domain showed that distributional measures outperform knowledge based path finding methods; but more recent studies suggested that intrinsic IC based measures exceed the accuracy of distributional approaches. Limitations of previous evaluations of similarity measures in the biomedical domain include their focus on the SNOMED CT ontology, and their reliance on small benchmarks not powered to detect significant differences between measure accuracy. There have been few evaluations of the relative performance of these measures on other biomedical knowledge sources such as the UMLS, and on larger, recently developed semantic similarity benchmarks.

**Results:**

We evaluated knowledge based and corpus IC based semantic similarity measures derived from SNOMED CT, MeSH, and the UMLS on recently developed semantic similarity benchmarks. Semantic similarity measures based on the UMLS, which contains SNOMED CT and MeSH, significantly outperformed those based solely on SNOMED CT or MeSH across evaluations. Intrinsic IC based measures significantly outperformed path-based and distributional measures. We released all code required to reproduce our results and all tools developed as part of this study as open source, available under http://code.google.com/p/ytex. We provide a publicly-accessible web service to compute semantic similarity, available under http://informatics.med.yale.edu/ytex.web/.

**Conclusions:**

Knowledge based semantic similarity measures are more practical to compute than distributional measures, as they do not require an external corpus. Furthermore, knowledge based measures significantly and meaningfully outperformed distributional measures on large semantic similarity benchmarks, suggesting that they are a practical alternative to distributional measures. Future evaluations of semantic similarity measures should utilize benchmarks powered to detect significant differences in measure accuracy.

## Introduction

Semantic similarity measures estimate the similarity between concepts, and play an important role in a variety of text processing tasks, including document classification [1-5], information extraction [6], information retrieval [7,8], word sense disambiguation [9,10], automatic spelling error detection and correction systems [11].

Similarity approaches utilized in the biomedical domain can be roughly divided into knowledge based and distributional based methods [12-14]. Knowledge based methods utilize pre-existing knowledge sources, including dictionaries, taxonomies, and semantic networks. Among the knowledge based approaches to which much effort in the biomedical domain has been dedicated are methods that utilize the taxonomic structure of a biomedical terminology to compute similarity; these include path finding measures and intrinsic information content (IC) measures [13-16]. Distributional methods utilize the distribution of concepts within a corpus in conjunction with a knowledge source to compute similarity; these include corpus IC and context vector methods [13]. Information content is a measure of concept specificity and is typically estimated from concept frequencies within a corpus (corpus IC). In contrast, intrinsic IC is an estimate of IC computed from the structure of a taxonomy. Because they do not rely on a corpus, knowledge based methods are more practical to compute than distributional methods. However, it is unclear if knowledge based methods are as accurate as distributional methods: evaluations in the biomedical domain that compare these methods were inconclusive.

In the biomedical domain, semantic similarity measures have been evaluated on the Systematized Nomenclature of Medicine-Clinical Terminology (SNOMED CT); Medical Subject Headings (MeSH); and the Unified Medical Language System (UMLS), a compendium of biomedical source vocabularies that includes SNOMED CT and MeSH [13,14,16-20]. Most of these evaluations were performed on a small benchmark of 29 SNOMED CT concept pairs.

In this study we aim to 1) evaluate the effect of biomedical knowledge source selection on semantic similarity measure accuracy, 2) clarify the impact of using the UMLS versus original source vocabularies, and 3) evaluate knowledge based measures and compare them to previously reported evaluations of distributional measures. We used larger, recently developed benchmarks that potentially have the power to detect significant differences between measures.

This paper is organized as follows: in the background section, we provide an overview of semantic similarity and relatedness measures, biomedical knowledge sources, and previous evaluations. In the methods section, we discuss our implementation of semantic similarity measures and the techniques used to evaluate them. In the results and discussion section, we present and discuss the results of evaluations on semantic similarity benchmarks.

## Background

### Semantic similarity and relatedness measures

Although technically they refer to different notions of relatedness, the terms similarity and relatedness are often used interchangeably [12]. This is in part due to the fact that these measures are applied to the same types of text processing tasks and evaluated on the same benchmarks [9,21]. Semantic similarity is a type of semantic relatedness, namely taxonomic relatedness, e.g. Lovastatin *is-a* Statin [22]. Semantic relatedness can refer to non-taxonomic relatedness such as antonymy, meronymy (*part-of*), frequent association, and other functional relationships (e.g. *treated-by*) [23].

Knowledge based semantic similarity measures include *random walk*, *path finding*, and *intrinsic IC* based measures. These measures generate a concept graph from a taxonomy or semantic network in which vertices represent concepts and edges represent semantic relationships. Path finding and intrinsic IC based measures utilize taxonomies, i.e. an acyclic, directed concept graph in which edges represent taxonomic relationships. A taxonomy suitable for use with semantic similarity measures can be derived from a knowledge source by taking a subset of hierarchical semantic relationships, and removing relations that induce cycles. Concepts that are generalizations of other concepts are referred to as parents or hypernyms; specifications of a concept are referred to as children or hyponyms.

Path finding based semantic similarity measures compute similarity as a function of the length of the shortest path between two concepts. One limitation of path finding measures is that they give equal weight to all relationships [13]. Information content (IC) based measures attempt to correct for this by weighting edges based on IC, a measure of concept specificity [13,14,24-26]. Relationships between specific concepts, e.g. Lovastatin *is-a* Statin, should be weighted more heavily than relationships between general concepts, e.g. Lovastatin *is-a* Enyme Inhibitor. Intrinsic IC based measures compute the information content of concepts from the taxonomic structure. The assumption underlying this approach is that the taxonomic structure is organized in a meaningful way, such that concepts with many hyponyms and few hypernyms have lower IC [14,25].

Random walk measures compute the relatedness between a pair of concepts via random walks on a concept graph [21,27,28]. In contrast to path finding and intrinsic IC measures, random walk measures can utilize graphs that contain undirected edges, non-taxonomic relationships, and cycles. Instead of defining relatedness as a function of the shortest path between concepts, random walk methods measure the overall connectivity between concepts. We focus on the personalized PageRank (PPR) algorithm that achieved state of the art performance on general language semantic similarity benchmarks, but has not been evaluated on biomedical semantic similarity tasks [21]. For a given concept, PPR generates a score vector that represents its connectivity to other concepts. The relatedness between a pair of concepts is defined as the cosine of the angle between their score vectors.

Distributional based measures utilize a domain corpus in conjunction with a knowledge source; these include *corpus IC* and *context vector* measures. Corpus IC based methods are analogous to intrinsic IC based methods, but estimate the information content of a concept from its distribution in a corpus.

Context vector measures of semantic relatedness are based on the assumption that words that appear in similar contexts are related [13,29]. This approach starts by creating *word vectors* from a corpus that represent word co-occurrence. Then *descriptor terms* for a concept are derived from a knowledge source such as a dictionary or thesaurus, and can be further expanded to include descriptor terms from related concepts [13,30-32]. The word vectors corresponding to a concept’s descriptor terms are then aggregated to construct a *context vector*[13]. The similarity between a pair of concepts is defined as the cosine of the angle between their context vectors.

In the biomedical domain, a study by Pedersen et al. that utilized a large medical corpus to estimate concept distributions showed that distributional measures outperformed taxonomy based path finding measures [13]. A more recent study by Sanchez et al. showed that knowledge based intrinsic IC measures outperformed distributional measures [14]. However, methodological differences in this latter study prevent a direct comparison between knowledge based and distributional based measures.

Previous work in the general language domain suggests that distributional measures of similarity suffer from limitations that stem from the imbalance, sparseness, and textual ambiguity of corpora [23,33]. More recent work that utilized a large (1.6 terabytes) web corpus, processed using substantial computational resources (2000 CPU cores), demonstrated that utilization of large corpora enable distributional measures to overcome these limitations, allowing distributional measures to achieve the same performance as knowledge based measures [12].

However, it is not clear if these results obtained via distributional methods in the general language domain are applicable in the biomedical domain, due to the lack of large, publicly available clinical corpora [14,34]. Furthermore, the computational resources required to process large corpora pose a practical challenge to the implementation of distributional measures.

Evaluations of similarity measures in the biomedical domain used private, institution-specific corpora of clinical notes [13,32]. Large corpora of clinical notes are not publicly available due to the sensitivity of the data contained therein, and smaller, publicly available corpora may bias concept frequency estimates [35]. Alternatively, it is possible to utilize a publicly available biomedical corpus such as MEDLINE, which contains over 19 million abstracts from biomedical journals [36]. However, evaluations of context vector measures based on 300,000 MEDLINE abstracts demonstrated poorer performance than measures based on a clinical corpus [32]. Using a larger subset of MEDLINE may overcome this problem, but processing this corpus represents a technical challenge that may be prohibitive for many applications. Furthermore, to compute corpus IC-based measures, text must be mapped to concepts. Automated concept mapping errors may bias concept frequency counts, negatively impacting the accuracy of corpus IC based measures [14].

### Biomedical knowledge sources – SNOMED CT, MeSH, UMLS

SNOMED CT is a comprehensive clinical ontology maintained by the International Health Terminology Standards Development Organisation (IHTSDO). MeSH is a controlled vocabulary thesaurus maintained by the National Library of Medicine (NLM) and used to index articles for the MEDLINE database. The UMLS Metathesaurus is a compendium of over 168 biomedical vocabularies including SNOMED CT and MeSH [37]. In this paper, when we refer to the UMLS, we are in fact referring to the UMLS Metathesaurus. All of these knowledge sources assign concepts unique identifiers, associate concepts with lexical variants (synonyms), and define hierarchical *is-a* relationships between concepts. SNOMED CT and the UMLS also enumerate additional semantic relationships, e.g. *part-of* and *treated-by*.

Advantages of the UMLS with respect to SNOMED CT or MeSH include robust tool support and broader concept coverage. Several popular natural language processing (NLP) tools map free text to UMLS concepts, facilitating the application of similarity measures based on the UMLS [38,39]. If a concept is missing from a knowledge source, then it is not possible to compute its similarity: use of multiple UMLS source vocabularies enables the computation of similarity measures not possible with a single source such as SNOMED CT or MeSH.

However, the UMLS introduces potential problems that may affect the accuracy of semantic similarity measures. The UMLS representation of source vocabularies may change concept granularity and/or distort relationships between concepts, thereby negatively impacting semantic similarity measures. For example, SNOMED CT distinguishes between morphological abnormalities and clinical findings: the Glomus tumor (morphologic abnormality) and Glomus tumor (disorder) represent distinct concepts in SNOMED CT; in the UMLS, these represent the same concept [40]. Clarifying the impact of using the UMLS versus original source vocabularies is one of the goals of this study.

### Semantic similarity benchmarks

Previous comparisons of semantic similarity and relatedness measures in the biomedical domain were performed on a benchmark of 29 SNOMED CT concept pairs created by Pedersen et al. (referred to as the Pedersen benchmark) [13]. Nine medical coders and three physicians assessed the semantic relatedness of these medical concept pairs. Pedersen et al. evaluated similarity measures against similarity scores for the coders, physicians, and the average of scores between groups (‘combined’); on this evaluation, distributional measures achieved the highest correlation with human raters.

Subsequent evaluations of semantic similarity measures in the medical domain utilized Pedersen’s benchmark. However, these comparisons may be flawed due to the difference in SNOMED CT versions used, and differences in the methods used to compute correlation between similarity measures and the reference standard: in their original study Pedersen et al. used SNOMED CT 2004 and the non-parametric Spearman rank test to measure the correlation between semantic similarity measures and the reference standard, as annotators rated concept similarity on an ordinal scale [13]. Subsequent studies used later SNOMED CT versions, and used the Pearson correlation coefficient, which is not comparable to the Spearman rank correlation coefficient [14,15,17]. In addition, the limited size of this reference standard may lack the power to detect significant differences between different measures.

Recently, Pakhomov et al. developed larger benchmarks for semantic relatedness and similarity using UMLS medical concept pairs. In the ‘Mayo’ benchmark, the same 9 medical coders and 4 physicians that supplied ratings for the Pedersen benchmark rated a set of 101 UMLS concept pairs for semantic relatedness on an ordinal scale [41]. In the ‘UMN’ benchmark, eight medical residents ranked a set of 587 and 566 UMLS concept pairs on a continuous scale for relatedness and similarity respectively [19]. Because the UMN ratings follow a multi-modal distribution, Pakhomov et al. used the Spearman rank correlation to evaluate semantic similarity and relatedness measures.

In the present study, in addition to the Pedersen benchmark, we evaluated semantic similarity measures on the Mayo and UMN benchmarks as they potentially have the power to detect significant differences in the accuracy of different measures.

## Methods

### Semantic similarity measures

In this section, we define the semantic similarity measures evaluated in this study. We modified some measures so that they conform to the universal definition of similarity presented by Lin [26]: measures are limited to the interval [0,1], and the similarity between a concept and itself is 1.

#### Path finding measures

We focus on the *Path*[13]*, Leacock & Chodorow* (LCH) [42]*,* and *Wu & Palmer*[43] path finding measures that are based on the shortest path separating concepts. Let *p=path*(*c*_*1*_*, c*_*2*_), the number of nodes in the shortest path separating two concepts, *c*_*1*_ and *c*_*2*_. The shortest path between two concepts traverses their Least Common Subsumer (*lcs*(*c*_*1*_*, c*_*2*_)), i.e. their closest common parent. The depth (*depth*(*c*)) of a concept is defined as the number of nodes in the path to the root of the taxonomy; and *d* represents the maximum depth of a taxonomy.

*Path* defines the similarity between two concepts simply as the inverse of the length of the path separating them [13]:

(1) $$sim_{\mathrm{path}}\left(c_{1},c_{2}\right)=1/p$$

LCH is based on the ratio of path length to depth, but performs a logarithmic scaling [42]. Originally, LCH was defined as

(2) $$sim_{\mathrm{lch}}^{\mathrm{unscaled}}\left(c_{1},c_{2}\right)=-\log\left(p/2d\right)=\log\left(2d\right)-\log\left(p\right)$$

As proposed in [4], we scale LCH to the unit interval by dividing by log(2*d*). Dividing by a constant value has no effect on the spearman correlation with benchmarks: the relative ranks of concept pair similarities remain the same.

(3) $$sim_{\mathrm{lch}}\left(c_{1},c_{2}\right)=1-\frac{\log\left(p\right)}{\log\left(2d\right)}$$

*Wu & Palmer* scales the depth of the LCS by the length of the path between two concepts [43]:

(4) $$sim_{\mathrm{wp}}^{\mathrm{unscaled}}\left(c_{1},c_{2}\right)=\frac{2\times depth\left(lcs\left(c_{1},c_{2}\right)\right)}{path\left(c_{1},lcs\left(c_{1},c_{2}\right)\right)+path\left(c_{2},lcs\left(c_{1},c_{2}\right)\right)+2\times depth\left(lcs\left(c_{1},c_{2}\right)\right)}$$

One problem with this definition is that the similarity of a concept with itself is less than 1 (if *c*_*1*_ = *c*_*2,*_ then *path*(*c*_1_, *lcs*(*c*_1_, *c*_2_)) + *path*(*c*_2_, *lcs*(*c*_1_, *c*_2_)) = 2). Instead, we adopt the definition of Wu & Palmer used in the Natural Language Toolkit [44]:

(5) $$sim_{\mathrm{wp}}\left(c_{1},c_{2}\right)=\frac{2\times depth\left(lcs\left(c_{1},c_{2}\right)\right)}{p-1+2\times depth\left(lcs\left(c_{1},c_{2}\right)\right)}$$

Under this definition, if *c*_*1*_ = *c*_*2*_, then *p*-1 = 0, and the similarity measure evaluates to 1.

#### IC based measures

Information content can be estimated solely from the structure of a taxonomy (intrinsic IC), or from the distribution of concepts in a text corpus in conjunction with a taxonomy (corpus IC) [14,24,25].

The corpus IC (*IC*_*corpus*_*(c)*) of a concept is defined as the inverse of the log of the concept’s frequency [24]. The frequency of a concept is recursively defined using a taxonomy: it is based on the number of times the concept *c* occurs within a corpus (*freq*(*c*, *C*)), together with the number of times its children occur:

(6) $$IC_{\mathrm{corpus}}\left(c\right)=-\log\left(freq\left(c\right)\right)$$

(7) $$freq\left(c\right)=freq\left(c,C\right)+\sum_{c_{s}\in children\left(c\right)}freq\left(c_{s}\right)$$

We follow the intrinsic IC definition proposed by Sanchez et al. [14]:

(8) $$IC_{\mathrm{intrinsic}}\left(c\right)=-\log\left(\frac{\frac{\left|\text{leaves}\left(c\right)\right|}{\left|\text{subsumers}\left(c\right)\right|}+1}{\text{max\textbackslash{}\_leaves}+1}\right)$$

where *leaves(c)* is the set of leaves (concepts without children) that are descendants of the concept *c*; *subsumers(c)* contains *c* and all its ancestors. The ratio of leaves to subsumers quantifies the information a concept carries– the more leaves a concept has relative to the number of ancestors, the less information it carries; this is normalized to the unit interval by *max_leaves*, the total number of leaves in the taxonomy.

The IC based Lin measure compares the IC of a concept pair to their LCS’s IC: the greater the LCS’s IC (i.e. the more specific the LCS), the more ‘similar’ the pair of concepts.

(9) $$sim_{\mathrm{lin}}\left(c_{1},c_{2}\right)=\frac{2\times IC\left(lcs\left(c_{1},c_{2}\right)\right)}{IC\left(c\right)+IC\left(c_{2}\right)}$$

Sanchez & Batet redefined path finding measures in terms of information content [14]. Path finding measures are defined in terms of the path length *p* and the maximum depth *d*. Sanchez & Batet proposed redefining the maximum depth d as *ic*_*max*_, the maximum information content of any concept; and proposed redefining the minimum path length *p* between two concepts in terms of Jiang & Conrath’s semantic distance [14,45]:

(10) $$dist_{\mathrm{jc}}\left(c_{1},C_{2}\right)=IC\left(c_{1}\right)+IC\left(c_{2}\right)-2\times IC\left(lcs\left(c_{1},c_{2}\right)\right)$$

The IC-based *LCH* measure is obtained simply by substituting *dist*_*jc*_ and *ic*_*max*_ for *p* and *d* in equation 3 (1 is added to *dist*_*jc*_ to avoid taking the logarithm of 0):

(11) $$sim_{\text{lch\textbackslash{}\_ic}}^{*}\left(c_{1},c_{2}\right)=1-\frac{\log\left(dist_{\mathrm{jc}}\left(c_{1},c_{2}\right)+1\right)}{\log\left(2\times ic_{\max}\right)}$$

One problem with this definition is that the IC-based LCH can assume negative values. We modify this as follows:

(12) $$sim_{\text{lch\textbackslash{}\_ic}}\left(c_{1},c_{2}\right)=1-\frac{\log\left(dist_{\mathrm{jc}}\left(c_{1},c_{2}\right)+1\right)}{\log\left(2\times ic_{\max}+1\right)}$$

Both Sanchez & Batet’s and our definitions of the IC-based LCH are monotonically decreasing functions of *dist*_*jc*_, and thus produce identical spearman correlations with benchmarks.

The IC-based *Path* measure is obtained simply by substituting *dist*_*jc*_ for *p* (1 is added to *dist*_*jc*_ to avoid dividing by 0):

(13) $$sim_{\text{path\textbackslash{}\_ic}}\left(c_{1},c_{2}\right)=\frac{1}{dist_{\mathrm{jc}}\left(c_{1},c_{2}\right)+1}$$

#### Personalized PageRank

The PageRank algorithm, originally developed to rank web pages, is a general method for the ranking of vertices in a graph based on importance [46,47]. The PageRank algorithm models web surfing as a markovian process, with a user randomly jumping across links (edges) to other pages (vertices). The PageRank algorithm produces a probability distribution over vertices (probability vector): it assigns to each vertex a score that represents the portion of time a random surfer will spend there. The personalized PageRank algorithm biases random jumps toward a set of vertices [48]. In its application to semantic relatedness, a graph is created in which vertices represent concepts and undirected edges represent semantic relationships [21]. For a given concept, a probability vector is computed via the personalized PageRank algorithm with random jumps biased towards the given concept. The relatedness between two concepts is defined as the cosine of the angle between their probability vectors.

The Personalized PageRank algorithm models a knowledge source as a graph *G* with n vertices *v*_*1*_*…v*_*n*_ corresponding to concepts, and undirected edges between concepts that represent semantic relationships [21]. Let *d*_*i*_ be the outdegree of node *i*. Let M be a *n*x*n* transition probability matrix, where M_*ji*_ = 1/*d*_*i*_ if a link from i to j exists, and 0 otherwise. The PageRank vector P is obtained by resolving the following equation:

(14) $$P=cMP+\left(1-c\right)v$$

In the PageRank algorithm the vector *v* is an *n*x1 vector whose elements are 1/*n*, and *c* is the damping factor, a scalar value in [0,1]. The first term *cMP* represents navigation across the edges of the graph, and the second term (1-*c*)*v* represents the probability of jumping to any vertex. The damping factor weights the combination of these terms; we used the default damping factor of 0.85.

In the personalized PageRank (PPR) algorithm, probability mass is concentrated on a set of entries in the vector *v*, biasing the jumps towards certain vertices [48]. To compute the relatedness between a pair of concepts, for each concept, the vector *P* is computed using the PPR algorithm with *v*_*i*_ = 1 for the corresponding concept, and 0 otherwise. The relatedness of a pair of concepts is defined as the cosine of the angle between their PPR vectors.

### Evaluation method

#### Concept graph construction

We evaluated measures using current knowledge source releases: the July 2011 International Release of SNOMED CT; the 2012 MeSH descriptors and supplementary concept records; and the UMLS release 2011AB using a ‘default’ Metathesaurus installation with SNOMED CT and all restriction-free (level 0) source vocabularies; this includes MeSH and 60 other vocabularies. In this paper, when we refer to ‘*the* UMLS’ we are in fact referring to this default subset of the UMLS.

We compared similarity measures that utilize concept graphs derived from SNOMED CT, MeSH, the UMLS SNOMED CT and MeSH source vocabularies, and the entire UMLS. For each knowledge source, we constructed a taxonomy for use with semantic similarity measures; computed the depth and intrinsic IC of each concept; and implemented semantic similarity measures. We used *is-a* relationships in taxonomies derived from SNOMED CT; we utilized all hierarchical relationships in taxonomies derived from the MeSH and the UMLS.

To evaluate the PPR, we constructed two types of undirected concept graphs: graphs that used only taxonomic relationships (*PPR-taxonomy*), and graphs that used all relationships from the respective knowledge sources (*PPR-all*). One major advantage of the PPR method is its ability to leverage non-taxonomic relationships to compute concept *relatedness*. Evaluating the PPR on both types of concept graphs allows us to quantify the contribution of non-taxonomic relationships to the computation of concept relatedness. Refer to Additional file 1: Appendix 1 for a detailed description of concept graph construction.

We evaluated measures on the Pedersen, Mayo, and UMN similarity and relatedness benchmarks [13,19,41]. We also evaluated measures on a ‘high agreement’ subset of the UMN relatedness benchmark: term pairs from this subset had a inter-class correlation coefficient of 0.73 or greater, and had a distribution of scores and broad semantic types similar to the entire set [32].

#### Comparison between SNOMED CT/MeSH and their UMLS representations

To determine the effect of the UMLS representation on similarity measure accuracy, we evaluated similarity measures on concept graphs derived from SNOMED CT, MeSH, and the UMLS SNOMED CT and MeSH source vocabularies. We evaluated measures based on SNOMED CT on the Pedersen benchmark only. We did not evaluate measures based on SNOMED CT on the Mayo and UMN benchmarks, as the SNOMED CT concept mappings for the term pairs from these benchmarks are not available. These benchmarks provide UMLS concept mappings, and a single UMLS concept may map to multiple SNOMED CT concepts.

#### Effect of source vocabulary selection

To determine the effect of UMLS source vocabulary selection on similarity measures, we evaluated similarity measures derived from concept graphs constructed from the UMLS SNOMED CT source vocabulary, UMLS SNOMED CT + MeSH source vocabularies, and entire UMLS. We evaluated measures for these concept graphs on the Pedersen, Mayo, and UMN benchmarks.

#### Corpus vs. Intrinsic IC

We compared the Lin measure using both the intrinsic IC and corpus IC on taxonomies derived from MeSH and its UMLS representation. We used MeSH for the comparison of intrinsic to corpus IC due to the availability of concept frequencies: the 2012 MEDLINE/PubMed Baseline Repository (MBR) provides the frequencies of MeSH headings used to index MEDLINE articles. The 2012 baseline contains frequencies from over 20 million citations [49]. We used these frequencies to compute the corpus IC for all MeSH concepts as defined in equation 6.

We adapted the Pedersen, UMN, and Mayo benchmarks for use with MeSH and its UMLS representation. Several concepts from the Pedersen benchmark are missing from the MeSH vocabulary. To enable correlation with semantic similarity measures based on MeSH, we used the ‘closest’ corresponding MeSH header; e.g. we used the MeSH header for ‘knee’ in place of ‘knee meniscus’. We used subsets of the UMN and Mayo benchmarks for which both members of the concept pair are found in the MeSH. There is a many-to-one correspondence between concepts from the UMLS MeSH source vocabulary and MeSH descriptors; this allows the unambiguous mapping of UMLS concepts from the UMN and Mayo benchmarks to MeSH descriptors.

We used different concept mappings for the Pedersen benchmark and subsets of the UMN and Mayo benchmarks, therefore the MeSH correlations are not directly comparable to correlations obtained using SNOMED CT or other UMLS source vocabularies. Refer to Additional file 1: Appendix 1 for a detailed listing of term to concept mappings used for these benchmarks.

#### Statistical analysis

We assessed accuracy using the non-parametric spearman rank, which computes the correlation ρ between two random variables from their relative ranks. The path finding *LCH* and *Path* are monotonically decreasing functions of the shortest path between concepts and therefore produce the same relative ranks and are thus identical for the purposes of evaluating their correlation. We applied the Fisher r-to-z transformation to test the significance of difference in correlation between different measures and concept graphs, and to compare our results to previously published results obtained using distributional measures. The null hypothesis is that there is no significant difference in correlation between different measures. The probability of rejecting the null hypothesis when it is in fact false – the statistical power – is higher on the larger UMN benchmarks. We used R version 2.10.1 for all statistical calculations. We released all code and scripts required to reproduce our results as open source.

## Results

### Concept graph dimensions

Concepts from the UMLS SNOMED CT source vocabulary are in general more coarse-grained than SNOMED CT concepts; thus, the taxonomy derived from the UMLS SNOMED CT source vocabulary is smaller than the taxonomy derived from SNOMED CT (Table 1). In contrast, concepts from the UMLS MeSH source vocabulary are more fine-grained than MeSH headings: the taxonomy derived from the UMLS MeSH source vocabulary is larger than the taxonomy derived from MeSH. Combining the UMLS SNOMED CT and MeSH source vocabularies (sct-msh) is almost equivalent to the sum of the source vocabularies (sct-umls, msh-umls), indicating that there is little overlap between these source vocabularies. The combination of all UMLS source vocabularies results in a taxonomy that is substantially larger than concept graphs based solely on SNOMED CT and/or MeSH.

**Table 1:** Concept graph dimensions

**Name**	**Description**	**Taxonomy**		**PPR concept graph**	
		**Concepts**	**Relations**	**Concepts**	**Relations**
sct	SNOMED CT	295,700	440,641	295,701	869,962
sct-umls	UMLS SNOMED CT source vocabulary	284,213	431,393	319,824	1,272,567
msh	MeSH	232,290	331,345	232,290	331,234
msh-umls	UMLS MeSH source vocabulary	315,081	426,139	321,306	1,266,235
sct-msh	UMLS SNOMED CT and MeSH source vocabularies	588,153	953,213	615,845	2,528,089
umls	All UMLS Level 0 source vocabularies and SNOMED CT	1,861,805	2,580,066	2,046,351	7,876,264

The taxonomies include only those concepts that partake in taxonomic relationships. All concepts in the SNOMED CT and MeSH knowledge sources partake in taxonomic relationships; thus the concept coverage for the PPR graphs is identical to that of the corresponding taxonomies. On the UMLS, the PPR concept graphs that utilize all relationships from the UMLS have broader concept coverage than taxonomies constructed from the UMLS.

### Semantic similarity measure evaluation

We present the correlation for each combination of reference standard, concept graph (*sct, sct-umls*, *sct-msh*, *umls*), and measure in Table 2. We present the correlation for each combination of reference standard and measure for the concept graphs derived from MeSH and its UMLS representation (*msh*, *msh-umls*) in Table 3. We also include the results of previous evaluations, where they are comparable. We present the significance of the difference between the intrinsic IC based *LCH* (intrinsic *LCH*) and path finding based *LCH*. Refer to Additional file 2: Appendix 2 for a listing of the significance of differences between all pairs of measures, and between concept graphs.

**Table 2:** Comparison of correlations across measures and reference standards

**Benchmark**	**Concept graph**	**Knowledge based**							**Distributional**	
		**Path**		**Intrinsic IC**			**PPR**			
		**Wu & Palmer**	**Path LCH**	**Lin**	**Path**	**LCH**	**Taxonomy**	**All**	**Lin**	**Vector**
**Pedersen Coders N=29**	Pedersen 2006 [13]		0.51						0.75	0.75
	sct	0.66	0.66	0.61	0.60	0.61	0.61	0.66		
	sct-umls	0.56	0.54	0.49	0.45	0.45	0.70	^+^0.26		
	sct-msh	0.64	0.76	0.59	0.58	0.61	0.75	0.46		
	umls	0.74	0.65	0.70	0.69	0.69	0.76	0.73		
**Pedersen Physicians N=29**	Pedersen 2006		0.36						0.60	0.84
	sct	0.54	0.50	0.52	0.49	0.49	0.49	0.62		
	sct-umls	0.44	0.38	0.41	^+^0.35	^+^0.35	0.56	^+^0.19		
	sct-msh	0.57	0.62	0.53	0.52	0.53	0.60	0.43		
	umls	0.66	0.60	0.72	0.69	0.69	0.67	0.63		
**Pedersen Combined N=29**	Pedersen 2006		0.48						0.69	0.76
	sct	0.59	0.56	0.56	0.53	0.54	0.55	0.67		
	sct-umls	0.49	0.44	0.45	0.38	0.38	0.63	^+^0.20		
	sct-msh	0.62	0.69	0.57	0.56	0.57	0.66	0.45		
	umls	0.70	0.61	0.72	0.70	0.70	0.69	0.68		
**Mayo N=101**	Pakhomov 2011 [41]	0.30	0.29							
	sct-umls	^+^0.05	^+^0.03	^+^0.09	^+^0.12	*0.30	^+^0.17	^+^0.00		
	sct-msh	0.28	0.22	0.32	0.33	0.35	0.44	^+^0.13		
	umls	0.38	0.30	0.39	0.41	0.44	0.46	0.21		
**UMN similarity N=566**	Pakhomov 2010 [19]		0.14							0.02
	sct-umls	0.21	0.23	0.22	0.23	**0.36	0.23	^+^0.00		
	sct-msh	0.30	0.30	0.32	0.32	**0.37	0.33	0.07		
	umls	0.39	0.40	0.43	0.43	0.46	0.41	0.25		
**UMN relatedness N=587**	Pakhomov 2010		0.10							−0.13
	sct-umls	0.14	0.17	0.16	0.16	**0.30	0.17	−0.01		
	sct-msh	0.21	0.20	0.22	0.23	**0.31	0.23	^+^0.04		
	umls	0.32	0.34	0.35	0.35	0.39	0.33	0.18		
**UMN relatedness subset N=430**	Liu 2010 [32]									0.46
	sct-umls	0.13	0.17	0.16	0.16	**0.30	0.17	^+^0.03		
	sct-msh	0.20	0.20	0.22	0.23	*0.32	0.23	^+^0.05		
	umls	0.33	0.36	0.36	0.36	0.40	0.35	0.22		

+Correlation not significant at 0.05 level. Significance of difference between Intrinsic LCH and Path Finding LCH **<0.05, *< 0.20. Abbreviations: LCH - Leacock & Chodorow, PPR – Personalized PageRank. Refer to Table 1 for concept graph descriptions.

**Table 3:** Comparison of correlations across measures and reference standards with MeSH

**Benchmark**	**Concept Graph**	**Knowledge Based**							
		**PPR**		**Path finding**		**Intrinsic IC**			**Corpus IC Lin**
		**Taxonomy**	**All**	**Wu & Palmer**	**Path, LCH**	**Path**	**LCH**	**Lin**	
**Pedersen Coders N=29**	msh	0.36		0.42	0.42	0.51	0.54	0.51	0.51
	msh-umls	0.20	0.20	0.37	0.38	0.44	0.53	0.45	0.43
**Pedersen Physicians N=29**	msh	0.30		0.40	0.40	0.41	0.39	0.41	0.41
	msh-umls	0.15	0.15	0.40	0.41	0.42	0.41	0.42	0.38
**Pedersen Combined N=29**	msh	0.31		0.42	0.42	0.46	0.43	0.46	0.46
	msh-umls	0.16	0.16	0.41	0.42	0.44	0.45	0.45	0.41
**Mayo N=61**	msh	0.27		0.37	0.35	0.47	0.37	0.46	0.45
	msh-umls	0.05	0.05	0.20	0.26	0.25	0.25	0.25	0.20
**UMN similarity N=429**	msh	0.27		0.25	0.26	0.32	0.29	0.33	0.33
	msh-umls	0.18	0.18	0.26	0.25	0.29	0.29	0.30	0.29
**UMN relatedness N=432**	msh	0.35		0.33	0.33	0.41	0.36	0.41	0.42
	msh-umls	0.18	0.18	0.35	0.34	0.34	0.35	0.34	0.34

Abbreviations: LCH - Leacock & Chodorow, PPR – Personalized PageRank. Refer to Table 1 for concept graph descriptions.

#### Differences between knowledge based measures

In general, intrinsic IC based measures outperformed path finding measures. On the larger Mayo and UMN reference standards, intrinsic IC based measures significantly outperformed path finding measures. The intrinsic IC based LCH measure achieved the best performance, but the improvement relative to other intrinsic IC based measures was usually not statistically significant (see Additional file 2: Appendix 2 for p-values).

In general, the PPR measure computed with all relationships (PPR – all) achieved poor performance, and was significantly outperformed by path and intrinsic IC based measures. The PPR measure computed with taxonomic relationships (PPR – taxonomy) significantly outperformed path-based measures on the Mayo reference standard, and significantly outperformed intrinsic IC based measures on the Pedersen coders reference standard. On larger reference standards, this relationship was reversed: intrinsic IC based measures significantly outperformed PPR-taxonomy based measures on the Mayo and UMN datasets.

#### Effect of UMLS representation

Measures based on concept graphs derived from SNOMED CT and MeSH outperformed their respective UMLS representations (*sct* vs. *sct-umls*, *msh* vs. *msh-umls*). We evaluated SNOMED CT only on the Pedersen benchmark, in which the difference was significant only for PPR using all relationships (Table 2, PPR-all, p-values in Additional file 2: Appendix 2). We evaluated MeSH and its UMLS representation on all benchmarks (Table 3, p-values in Additional file 2: Appendix 2). The difference in performance between measures based on MeSH and its UMLS representation was significant for some benchmark/measure combinations.

#### Effect of UMLS source vocabulary selection

Increasing the concept graph size improved the performance of both path finding and intrinsic IC based measures: measure performance increased with the size of the concept graph. Measures based on the concept graph derived from the entire UMLS achieved the best performance, and this difference was statistically significant and meaningful (Table 4).

**Table 4:** Significance of differences in Intrinsic LCH correlation between taxonomies

**Benchmark**	**Concept graphs**	
	**sct-umls vs umls**	**sct-msh vs umls**
Pedersen Coders		
Pedersen Physicians	*	
Pedersen Combined	*	
Mayo		
UMN similarity	*	*
UMN relatedness	*	*

Significance of difference between Intrinsic IC based LCH on different concept graphs *< 0.20. Refer to the Additional file 2: Appendix 2 for p-values.

#### Knowledge vs. distributional based methods

##### Pedersen

On the Pedersen coders and combined benchmarks, there is no significant difference between the best knowledge and distributional based measures (Table 2, Coders 0.76 vs. 0.75, Combined 0.72 vs 0.76 ); on the Pedersen Physicians benchmark, the context vector measure outperforms the best knowledge based measure (0.72 vs 0.84, p-value=0.18).

##### *UMN.*

All knowledge based measures significantly outperformed the context vector measure on the UMN similarity and relatedness benchmarks (Table 2, UMN similarity and relatedness benchmarks, p-value=0 on *umls* concept graph). Pakhomov et al. evaluated the context vector measure on the UMN benchmark [19]. The context vector utilized a co-occurrence matrix derived from 500,000 EMR inpatient reports and had correlations of 0.02 and −0.13 with the UMN similarity and relatedness benchmarks respectively; for comparison, the worst-performing path-based measures from our evaluations had correlations of 0.21 and 0.14 respectively.

##### *UMN Relatedness Subset.*

Liu et al. evaluated the context vector measure on a subset of the UMN benchmark [32]. Although they achieved a higher correlation, the difference to the best knowledge based measure was not statistically significant (Table 2, UMN relatedness subset, 0.46 vs 0.40).

##### *Intrinsic IC vs. Corpus IC.*

We evaluated the corpus and intrinsic IC based Lin measure using MeSH. There was no significant difference in correlation between the intrinsic and corpus IC based measures on any reference standard. In general, the intrinsic IC based Lin outperformed corpus IC based Lin on UMLS MeSH taxonomy (*msh-umls*); however, these differences were not statistically significant (refer to Additional file 2: Appendix 2 for p-values).

### System performance and interoperability

The system we developed is open source and written in the platform independent Java language. It is a generalizable framework for the computation of semantic similarity measures from any taxonomy or semantic network; in this study we utilized SNOMED CT, MeSH, and the UMLS Metathesaurus. The system allows the declarative definition of concept graphs or taxonomies and stores these graphs in a binary format. For taxonomies, it computes the depth and intrinsic information content of each node. The system provides programmatic, command line, RESTful, and XML web services interfaces to users to compute similarity measures. We provide a publicly available web service to compute semantic similarity measures. Notable aspects of our system include the ability to compute both intrinsic IC and corpus IC based measures, and the ability to compute similarity measures from a wide range of biomedical knowledge sources. The pure java implementation simplifies the integration of our system with popular java based text processing frameworks such as the Unstructured Information Management Architecture (UIMA) and the General Architecture for Text Engineering (GATE) [50,51].

The time and computational resources needed to generate concept graphs varies based on size. Computing the intrinsic information content is the most computationally and memory intensive step in preparing a taxonomy. This required less than 1 minute with 1GB of memory for a small concept graph such as SNOMED CT; for the entire UMLS, this required 90 minutes with 8GB of memory. Once created, the concept graph can be loaded and used to compute similarity measures. The time and resources needed to load the concept graph depends on its size; loading the taxonomy for the entire UMLS required 30 seconds and 1 GB of memory. All computations were performed on a 64-bit Ubuntu 10 Linux workstation with dual quad-core 3.00GHz Intel Xeon processors.

Computing path finding and IC based similarity measures on the UMN relatedness benchmark (n=582) with the UMLS taxonomy required 8 seconds (after initialization). The computation of relatedness via the personalized PageRank algorithm is computationally intensive, and increases with concept graph size. Computing PPR for the UMN relatedness benchmark with the UMLS concept graph required 5 hours.

## Discussion

### Effect of UMLS representation

Our results suggest that the UMLS representation of source vocabularies such as MeSH and SNOMED CT changes them in a manner that negatively impacts semantic similarity measure performance. However, the utilization of other UMLS source vocabularies in addition to the UMLS SNOMED CT and MeSH source vocabularies more than makes up for this: using multiple vocabularies enables broader concept coverage, and significantly improves the correlation of similarity measures with human judgments.

### Differences between knowledge based measures

Intrinsic IC based measures in general outperformed path based measures; in some cases, these differences were significant. Intrinsic IC and path based measures compute similarity as a function of the distance between concepts in a taxonomy. IC based measures achieve higher performance than path based measures by weighting taxonomic links based on concept specificity.

The personalized PageRank algorithm achieved state of the art performance on general language semantic similarity tasks, but did not outperform simpler knowledge based methods on these benchmarks. Furthermore, PPR is orders of magnitude more computationally intensive than simpler semantic similarity measures, and may be impractical for some applications. In contrast to other knowledge based similarity measures, PPR can utilize non-taxonomic relationships to compute concept relatedness. However, using non-taxonomic relationships significantly reduced PPR’s performance on these benchmarks. The UMLS contains many types of non-taxonomic relationships. It may be possible that using a subset of non-taxonomic relationships would improve PPR’s performance.

### Knowledge vs. distributional based measures

Our results suggest that knowledge based measures can outperform distributional measures. Knowledge based measures are also more practical than distributional measures, as they do not require a corpus from which word co-occurrence or concept frequencies must be estimated.

Knowledge based measures significantly and meaningfully outperformed distributional vector based measures on the larger UMN benchmarks. One limitation to our study is that we compared knowledge based methods to previously published distributional vector based measures: we cannot exclude the possibility that differences in the UMLS version used may have biased results. However, our reasons for not implementing context vector measures represent exactly their limitations: a large clinical corpus is not available to us; it is not clear if publicly available corpora such as MEDLINE abstracts are suitable for this purpose; and the processing of large corpora is computationally intensive.

Distributional vector based measures in the biomedical domain may suffer from imbalance and sparseness due to limited corpus sizes [23,33]. Use of a larger clinical corpus may rectify these issues, and improve the performance of vector based measures relative to knowledge based measures. Even if performance could be improved with a large corpus, it is not clear what practical consequences this would have, as many applications of semantic similarity measures lack access to large clinical corpora.

Our evaluation showed no significant differences between corpus IC and intrinsic IC based measures. We used MeSH for the comparison of intrinsic IC to corpus IC, and estimated corpus IC using the frequencies of MeSH headings derived from over 20 million MEDLINE abstracts. These results suggest that, given the ease with which IC can be estimated from a taxonomy, intrinsic IC based measures are a practical alternative to corpus IC based measures. One limitation of our study is that we only evaluated corpus IC based measures with MeSH using concept frequencies estimated from a biomedical corpus. Results obtained with SNOMED CT or the UMLS using concept frequencies from a clinical corpus may differ. However, for many applications, computing corpus IC may not be practical: in addition to the lack of availability of large clinical corpora, the estimation of concept frequencies requires an annotated corpus. Automated concept annotation methods may be confounded by textual ambiguity, and manual concept annotation may be impractical for large corpora [14].

### Future directions

Strengths of our study include the evaluation of a wide range of measures using multiple benchmarks and knowledge sources, and the assessment of the statistical significance of differences between measures and across knowledge sources. Previous evaluations of semantic similarity and relatedness in the biomedical domain utilized the Pedersen benchmark of 29 concept pairs with SNOMED CT. On the smaller Pedersen physicians benchmark, distributional vector based measures significantly outperformed knowledge based measures. In contrast, on the larger UMN benchmark, intrinsic IC based measures significantly outperformed path finding and distributional vector based measures. These findings suggest that future evaluations of semantic similarity and relatedness measures in the biomedical domain should utilize larger benchmarks to ensure the reliability of results.

To facilitate the application of semantic similarity measures to text processing applications, we developed tools for computing semantic similarity measures; we integrated these tools with a popular clinical natural language processing pipeline; and we released them as open source, available under http://code.google.com/p/ytex. We are currently evaluating semantic similarity measures on word sense disambiguation and document classification tasks.

## Conclusion

We evaluated knowledge based semantic similarity measures using different biomedical knowledge sources, and we compared the accuracy of these measures against benchmarks of semantic similarity and relatedness. We found that intrinsic IC based measures achieved the best performance across a wide range of benchmarks and knowledge sources; intrinsic IC based measures performed as well or better than distributional measures; and that measures based on the UMLS achieve significantly higher accuracy than those based on smaller knowledge sources such as MeSH or SNOMED CT.

## Competing interests

The authors declare that they have no competing interests.

## Authors' contributions

VG conceived the study, implemented the algorithms, and performed the evaluations. VG and CB drafted the original manuscript. All authors read and approved the final manuscript.

## Supplementary Material

Additional file 1 Appendix 1. Click here for file

Additional file 2 Appendix 2. Click here for file
