# Supplementary material for: Semantic similarity in the biomedical domain: an evaluation across knowledge sources
Source: BMC Bioinformatics. 2012 Oct 10;13:261. doi: 10.1186/1471-2105-13-261 (PMC3533586; doi:10.1186/1471-2105-13-261)
Supplement: Additional file 1 — Appendix 1. [file 1471-2105-13-261-S1.docx]

Appendix: Semantic Similarity

# Concept Graph Construction

Our system takes as input a set of directed edges and creates a concept graph. We utilize 2 types of concept graphs in this study: an undirected graph for use with PPR, and a taxonomy for use with semantic similarity measures.

To generate undirected graphs for use with PPR, we simply provide our system a set of directed edges that point in both directions, i.e. for vertices A, and B, if the graph contains an edge from A->B, then it also contains an edge from B->A.

The system can be configured to generate a taxonomy from a set of edges. A taxonomy is a directed, acyclic concept graph with a single node that has no parents (*the* root). To create a taxonomy, the system removes edges that induce cycles, and creates a root concept if required. The problem of obtaining acyclic graphs from a graph with cycles by removing edges is known as the feedback arc set problem and is NP-hard (1). We adopt a simple heuristic solution: as we build the graph, we check each edge to determine if it induces a cycle. If so, we discard the edge. To ensure that the taxonomies generated in this way are reproducible, we retrieve edges in a deterministic order.

When combining multiple UMLS Source Vocabularies, there can be multiple ‘candidate roots’, i.e. concepts without parents. In these situations, the system creates a single synthetic root, and adds the candidate roots as children of the synthetic root.

We also discard edges that contain ‘illegal concepts’ from a knowledge source; in the UMLS, these include e.g. C1274012 (ambiguous concept).

We imported the UMLS, SNOMED-CT, and MeSH into a MySQL v5.1 database. To generate concept graphs, we retrieved relationships from the database. We made available the SQL statements used to retrieve edges as part of our source archive, available under http://code.google.com/p/ytex.

## UMLS Concept Graphs

We installed the UMLS version 2011AB in a MySQL database, and retrieved relationships (edges) from the UMLS MRREL table to create concept graphs. The MRREL table contains the SAB and REL columns that contain the source vocabulary and relation type respectively. To create concept taxonomies, we retrieved edges from the MRREL table that had the SAB and REL specified below. To create concept graphs for use with the PPR measure, we retrieved edges from the MRREL table that had the SAB specified below, with no filtering on the REL field.

Table 1: MRREL filters

| Concept Graph | SAB | REL |
| --- | --- | --- |
| sct-umls | SNOMEDCT | PAR |
| msh-umls | MSH | PAR  RB |
| sct-msh | SNOMEDCT  MSH | PAR  RB |
| umls | All Level 0 vocabularies and SNOMEDCT | PAR  RB |

## SNOMED-CT Concept Graph

We downloaded the July 2011 International Release of SNOMED-CT release format 2 files and imported them into MySQL database tables, as documented in section 7.2.1.3.1 of the SNOMED technical implementation guide (2). We generated a taxonomy from all active *is-a* relationships. We generated a PPR concept graph from all active relationships (including *is-a* relationships).

## MeSH Concept Graph

MeSH enumerates descriptors (headers), and organizes these descriptors in a tree. MeSH also enumerates supplementary concepts that are mainly chemicals and specifies taxonomical links to descriptors, e.g. Garlic Oil *is-a* Allyl Compound. Our MeSH concept graph represents MeSH descriptors and supplementary concepts as vertices. The edges of our MeSH concept graph represent MeSH tree relationships and taxonomical links between MeSH supplementary concepts and descriptors.

We downloaded the 2012 MeSH descriptors and supplementary concept records, distributed as XML files. We parsed the XML files and imported descriptors, supplementary concepts, and taxonomic relationships into relational database tables. We retrieved edges for the construction of the MeSH taxonomy and PPR concept graph from the database. Code for parsing MeSH XML files, and the SQL statements for retrieving edges are included in the source archive.

## MeSH Concept Graph

The taxonomy derived from the UMLS MeSH representation (msh-umls) differs from the taxonomy derived from MeSH (msh); these differences may account for the significantly better performance of similarity measures based on *msh* vs. *msh-umls*. One major difference is that there is no single UMLS CUI for a given MeSH descriptor. MeSH links descriptors to one or more UMLS CUIs, and designates one of the CUIs a ‘preferred’ concept (Table 2).

Table 2: MeSH Descriptors and linked UMLS CUIs

| Descriptor | UMLS Concept | Description | Preferred |
| --- | --- | --- | --- |
| D010607 | C0031341 | Pharmacy Service, Hospital | Yes |
|  | C0008968 | Pharmacy Service, Clinical |  |
| C038491 | C0051235 | allyl sulfide | Yes |
|  | C1802750 | garlic oil |  |

The UMLS does not represent MeSH descriptors as concepts. For many descriptors, the preferred concept is designated the parent of other UMLS concepts linked to a MeSH descriptor; i.e. the UMLS defines a ‘subtree’ for each MeSH descriptor. For example, the UMLS MRREL table contains an RB/RN entry that designates C0008968 ‘Pharmacy Service, Clinical’ a parent of C0031341 ‘Pharmacy Service, Hospital’.

For some descriptors, no taxonomic relationships are defined between the CUIs linked to the descriptor; instead the UMLS specifies ‘other relationship’ (RO) relations between the CUIs linked to the descriptor. For example, the UMLS MRREL table contains an RO entry that designates a non-taxonomic relationship between C0051235 ‘allyl sulfide’ and C1802750 ‘garlic oil’.

The UMLS ‘orphans’ many MeSH concepts. Many UMLS MeSH CUIs are not children of any other UMLS concept; for example, the UMLS MRREL table does not enumerate any parents for the CUI C0078968 (MeSH descriptor D016056, ‘Immunodominant Epitopes’). When we build a taxonomy, we create a ‘synthetic’ root, and add all nodes without parents as children of the synthetic root. The roots of the msh and msh-umls taxonomies have 111 and 6668 direct children respectively.

Orphaning concepts alters path lengths and information content computations. D018984 ‘T-Lymphocyte Epitopes’ and D016056 ‘Immunodominant Epitopes’ are siblings in MeSH (path length 3); the paths between the corresponding CUIs in the UMLS MeSH taxonomy must traverse the root instead of their common parent, increasing the path length and reducing similarity estimates. In MeSH, D016056 ‘Immunodominant Epitopes’ is a child of D000939 ‘Epitopes’ – the leaves/frequencies of D016056 contribute to the intrinsic/corpus IC estimates for this MeSH subtree. However, the in the UMLS MeSH representation, the leaves/frequencies of the concepts associated with D016056 do not contribute to the IC computations of the appropriate subtree.

The way that the UMLS models MeSH descriptors, and the ‘orphaning’ of MeSH concepts in the UMLS representation may account for the significantly poorer performance of measures based on *msh-umls* vs *msh*. Refer to the msh-vs-umls spreadsheet in simbenchmark.xls for the p-values for the difference in measure performance on the *msh* vs *msh-umls* concept graphs.

# MeSH Corpus IC Computation

We used the MEDLINE Baseline Repository (MBR) to compute the corpus IC for the *msh* and *msh-umls* concept graphs. The MBR provides the ‘raw’ frequency of a MeSH descriptors, with which we recursively computed concept frequency and information content (equations 6 & 7 from main paper).

To use the MBR to compute corpus IC on the *msh-umls* concept graph, MeSH descriptors from the MBR must be mapped to UMLS concepts. MeSH descriptors can be mapped to one or more UMLS concepts. To compute corpus IC for the msh-umls concept graph, we duplicated MBR frequencies for a given MeSH descriptor for each UMLS concept. For example, the MeSH descriptor D008820 (‘Mice, Obese’) is mapped to the UMLS Concepts C0025933 (‘Mice, Obese’) and C0162418 (‘Mouse, Hyperglycemic’), and has a ‘raw’ frequency of 3342. We assigned both UMLS Concepts (C0025933, C0162418) a ‘raw’ frequency of 3342.

We also experimented with assigning the MBR frequency for a given MeSH descriptor to a single UMLS Concept (the ‘preferred’ UMLS Concept). This however resulted in lower performance (results not shown).

# Supplementary data

This section describes additional information contained in the semanticsim.xlsx workbook.

## Concept Graph Comparisons

### Sct-vs-umls

This spreadsheet contains the p-values for the significance of the difference in the correlation of measures between the *sct* and *sct-umls* concept graphs.

### msh-vs-umls

This spreadsheet contains the p-values for the significance of the difference in the correlation of measures between the *msh* and *msh-umls* concept graphs.

### simbenchmark-cg-significance

This spreadsheet contains the p-values for the significance of the difference in the correlation of the intrinsic IC based LCH measure between the *sct*, *sct-umls*, *sct-msh*, and *umls* concept graphs.

## Measure Comparisons

### simbenchmark-spearman

This spreadsheet lists the spearman correlation and p-value for each combination of benchmark, concept graph, and measure.

### simbenchmark-summary

This spreadsheet contains the same data as table 2 from the main paper. In addition it has columns for the significance of differences in correlation between pairs of measures, e.g. the column “WUPALMER.PATH” contains the p-value for the significance of the difference between the Wu-Palmer and Path measures (fisher r-to-z transformation).

### simbenchmark-msh-summary

This spreadsheet contains the same data as table 4 from the main paper.

## Semantic Similarity and Relatedness Benchmarks Term Mappings

### MiniMayoSRS

This spreadsheet contains the term pairs, concept ids, and ratings for the 29 concept pairs from the ‘Pedersen’ benchmark (3). It contains the following columns:

- Physicians/Coders: The average ratings from the physicians and coders respectively
- TERM1/TERM2: The term pairs
- sct1/sct2: The SNOMED-CT concept ids for the respective terms, provided to us by David Sanchez (personal communication).
- CUI1/CUI2: The UMLS CUIs for the respective terms, available from (4). We changed CUIs that did not match the corresponding SNOMED-CT concepts (sct1/sct2):
  - Replaced C0175895 with C1265880. C0175895 is mapped to an obsolete SNOMED-CT concept
  - Replaced C0027627 (metastasis) with C2939419 (tumor metastasis). C0027627 (metastasis) is not in the UMLS SNOMED-CT source vocabulary.
- mesh cui1/mesh cui2: The MeSH CUIs for the respective terms. Most of the CUIs are identical to the CUI1/CUI2 columns. However, some CUIs from the UMLS SNOMED-CT source vocabulary are not present in the MeSH source vocabulary:
  - Replaced C0156543 (abortion) with C0392535 (abortion induced)
  - Replaced C1265880 (calcification) with C0006660 (Physiological Calcification)
  - Replaced C0009814 (stenosis) with C1261287 (stenosis)
  - Replaced C0344375 (stomach cramp) with C0038354 (Stomach Diseases)
  - Replaced C0409974 (lupus) with C0024141 (Systemic Lupus Erythematosus)
  - Replaced C0702166 (acne) with C0001144 (acne)
  - Replaced C0333997 (Lymphoid hyperplasia) with C0221269 (Lymphoid Hyperplasia, Reactive)
  - Replaced C0034887 (Rectal Polyp) with C0009376 (Colonic Polyp)
  - Replaced C0224701 (Entire Knee Meniscus) with C0022742 (Knee)
- mesh1/mesh2: The MeSH Descriptor IDs for the respective terms. We used the UMLS mapping from CUIs to MeSH descriptor IDs.

### MayoSRS

This spreadsheet contains the UMLS CUIs and ratings for the 101 concept pairs from the ‘Mayo’ benchmark (5). We replaced the obsolete CUI C2267026 with C0360714 (Statin). We added columns for the MeSH descriptor IDs.

### UMNSRS_relatedness and UMNSRS_similarity

These spreadsheets contain the UMLS CUIs and ratings for the ‘UMN’ semantic relatedness and similarity benchmarks (6). We replaced the obsolete CUI C1998461 with C0020517 (allergic reaction). We added columns for the MeSH descriptor IDs.

## Similarity Measure Outputs

This *sim* and *sim-mesh* spreadsheets contain the similarity measures on each concept pair for each combination of benchmark and concept graph. For each concept pair, the Least Common Subsumer and Path used to compute similarity measures is listed.

# References

1. Eades P, Lin X, Smyth WF. A Fast Effective Heuristic For The Feedback Arc Set Problem. Information Processing Letters. 1993;47:319–23.

2. International Health Terminology Standards Development Organization. SNOMED CT Technical Implementation Guide January 2012 International Release [Internet]. Available from: http://ihtsdo.org/fileadmin/user_upload/doc/download/doc_TechnicalImplementationGuide_Current-en-US_INT_20120131.pdf

3. Pedersen T, Pakhomov SVS, Patwardhan S, Chute CG. Measures of semantic similarity and relatedness in the biomedical domain. J Biomed Inform. 2007 Jun;40(3):288–99.

4. Pedersen T, Pakhomov SVS. Medical Coders High Reliability Subset [Internet]. [cited 2012 Mar 14]. Available from: http://rxinformatics.umn.edu/data/MiniMayoSRS.csv

5. Pakhomov SVS, Pedersen T, McInnes B, Melton GB, Ruggieri A, Chute CG. Towards a framework for developing semantic relatedness reference standards. Journal of Biomedical Informatics [Internet]. 2010 Oct [cited 2011 Apr 13]; Available from: http://linkinghub.elsevier.com/retrieve/pii/S1532046410001565

6. Pakhomov S, McInnes B, Adam T, Liu Y, Pedersen T, Melton G. Semantic Similarity and Relatedness between Clinical Terms: An Experimental Study. AMIA Annu Symp Proc. 2010;2010:572–6.
